# High-quality genetic mapping with ddRADseq in the non-model tree *Quercus rubra*

**DOI:** 10.1186/s12864-017-3765-8

**Published:** 2017-05-30

**Authors:** Arpita Konar, Olivia Choudhury, Rebecca Bullis, Lauren Fiedler, Jacqueline M. Kruser, Melissa T. Stephens, Oliver Gailing, Scott Schlarbaum, Mark V. Coggeshall, Margaret E. Staton, John E. Carlson, Scott Emrich, Jeanne Romero-Severson

**Affiliations:** 1 Department of Biological Sciences, University of Notre Dame, Notre Dame, IN 46556 USA; 2 Department of Computer Science and Engineering, University of Notre Dame, Notre Dame, IN 46556 USA; 3 Internal Medicine, Northwestern Memorial Hospital, Chicago, IL 60611 USA; 4 School of Forest Resources and Environmental Science, Michigan Technological University, Houghton, MI 49931 USA; 5 Department of Forestry, Wildlife and Fisheries, University of Tennessee, Knoxville, TN 37996 USA; 6 School of Natural Resources, University of Missouri-Columbia, Columbia, MO 65211 USA; 7 Department of Entomology and Plant Pathology, University of Tennessee, Knoxville, TN 37996 USA; 8 Department of Ecosystem Science and Management, Penn State, University Park, State College, PA 16802 USA; 9 Hardwood Tree Improvement and Regeneration Center, USDA Forest Service Northern Research Station, West Lafayette, IN 47907 USA

**Keywords:** *Quercus rubra*, Sequencing depth, ddRADseq, Dense linkage mapping

## Abstract

**Background:**

Restriction site associated DNA sequencing (RADseq) has the potential to be a broadly applicable, low-cost approach for high-quality genetic linkage mapping in forest trees lacking a reference genome. The statistical inference of linear order must be as accurate as possible for the correct ordering of sequence scaffolds and contigs to chromosomal locations. Accurate maps also facilitate the discovery of chromosome segments containing allelic variants conferring resistance to the biotic and abiotic stresses that threaten forest trees worldwide. We used ddRADseq for genetic mapping in the tree *Quercus rubra*, with an approach optimized to produce a high-quality map. Our study design also enabled us to model the results we would have obtained with less depth of coverage.

**Results:**

Our sequencing design produced a high sequencing depth in the parents (248×) and a moderate sequencing depth (15×) in the progeny. The digital normalization method of generating a *de novo* reference and the SAMtools SNP variant caller yielded the most SNP calls (78,725). The major drivers of map inflation were multiple SNPs located within the same sequence (77% of SNPs called). The highest quality map was generated with a low level of missing data (5%) and a genome-wide threshold of 0.025 for deviation from Mendelian expectation. The final map included 849 SNP markers (1.8% of the 78,725 SNPs called). Downsampling the individual FASTQ files to model lower depth of coverage revealed that sequencing the progeny using 96 samples per lane would have yielded too few SNP markers to generate a map, even if we had sequenced the parents at depth 248×.

**Conclusions:**

The ddRADseq technology produced enough high-quality SNP markers to make a moderately dense, high-quality map. The success of this project was due to high depth of coverage of the parents, moderate depth of coverage of the progeny, a good framework map, an optimized bioinformatics pipeline, and rigorous premapping filters. The ddRADseq approach is useful for the construction of high-quality genetic maps in organisms lacking a reference genome if the parents and progeny are sequenced at sufficient depth. Technical improvements in reduced representation sequencing (RRS) approaches are needed to reduce the amount of missing data.

**Electronic supplementary material:**

The online version of this article (doi:10.1186/s12864-017-3765-8) contains supplementary material, which is available to authorized users.

## Background

The low cost and broad applicability of reduced representation sequencing (RRS) technologies have enabled a burst of genetic architecture and gene discovery studies in natural populations. A widely used RRS technique, restriction site associated DNA sequencing (RADseq), inexpensively generates tens of thousands of SNP calls, a seemingly sufficient number for detecting fine-scale population substructure, constructing phylogenies, and generating densely populated genetic maps [1–4]. Technical evaluations of RADseq show that library construction techniques, DNA quality, read coverage, and informatics strongly influence the accuracy and number of SNP calls but most of these studies are focused on the application of RADseq technologies for phylogenetic and population fine structure analyses [5–8]. SNP calling errors and missing data have different impacts on phylogenetic and population fine structure analyses than on the construction of densely populated genetic maps. In this study we tested the performance of double digest RADseq (ddRADseq) [9, 10] for dense genetic mapping in northern red oak (*Quercus rubra* L.), a highly heterozygous, outcrossing angiosperm forest tree lacking a reference genome. None of the ecologically dominant and economically valuable oaks of eastern North America have reference genomes and studies of population dynamics remain limited. RRS techniques have the potential to provide an affordable technology for dense genetic mapping and gene discovery in oaks and the other long-lived angiosperm forest trees of eastern North America.

Our approach to mapping with ddRADseq included a full-sib population from one seed parent and one pollen parent, a pedigree that provides more information for genetic mapping than a half-sib family of the same size. The outcrossing parents were expected to have many SNP loci heterozygous for the same SNP, enabling the construction of one map rather than separate male and female maps. Both parents have seed, bud and leaf morphologies consistent with those expected for *Q. rubra*. We used a high coverage design, devoting one lane of sequencing on an Illumina HiSeq for the two parents and five lanes for the progeny (50/lane), considerably fewer than the 96 individuals typically loaded in each lane for RADseq involving non-model organisms lacking reference genomes [11]. The informatics pipeline included two approaches for generating a *de novo* reference for *Q. rubra* and two SNP variant callers. We generated a statistically robust framework map with gSSR and EST-SSR markers, and then used ddRADseq to discover SNP markers for the same individuals. This study design enabled us to test the performance of ddRADseq for genetic mapping under optimized conditions and to model the results we would have received had we used an experimental design with less coverage per individual.

Oaks are outcrossing, diploid forest trees with relatively tractable genome sizes (~600–800 Mb) [12, 13] and a haploid chromosome number of 12 [14]. *Quercus rubra* is the most dominant and wide-ranging species of the *Lobatae*, a section of the *Quercus* genus containing nearly 100 species ranging from California to the Atlantic coast, north to northern Ontario and south across Mexico and Central America to northern Columbia [15]. The *Lobatae* are ecologically significant in many forest communities of eastern North America, occurring across a wide range of ecosystems, including dry savannahs, mesic bottomlands and upland forests [16]. Unlike the white oaks (*Quercus* section *Quercus*), which are native in North America, Europe, and Asia the red oaks are native only in the Americas [17]. The accidental importation of exotic pests, diseases, and weedy species combined with short-sighted management practices threaten the health of the oak forests worldwide [18]. The development of high-quality genetic maps and other genomics tools for oaks, in combination with sound management, will enable more effective and timely responses to these challenges.

Prior studies on woody perennials have used RADseq to examine gene flow among ecologically divergent species of *Populus* [19], adaptive evolution through interspecific hybridization in *Populus* [20], signatures of selection in buckthorn (*Frangula alnus*) [21], adaptation to aridity in *Eucalyptus tricocarpa* [22] and phylogeny across the *Quercus* genus [23]. Use of a hypomethylation-sensitive enzyme and messenger RNA sequencing (mRNAseq) has permitted RADseq marker development for the gigantic 16 Gb genome of Atlas cedar (*Cedrus atlantica*) [24]. However, there are few reported RADseq efforts for generating genetic maps in non-domesticated woody perennials. Recent reports of mapping in woody perennials include an interspecific cross of the jujube fruit *Ziziphus* Mill. [25], a cross of the European pear (*Pyrus communis* L.) and the Chinese pear (*Pyrus bretschneideri* Rehd.) [26], pomelo (*Citrus grandis* Osbeck) [27], kiwifruit (*Actinidia chinensis* Pl.) [28], red raspberry (*Rubus idaeus* L.) [29], foxtail pine (*Pinus balfouriana* Grev. & Balf.) [30] and the interspecific cross *Populus deltoides* Marsh x *P. simonii* [31]. Of these, only foxtail pine, a conifer, and the angiosperm *Populus* species are undomesticated.

In the Fagaceae (oaks, chestnuts, and beeches), genetic maps are reported for the European pedunculate oak (*Quercus robur* L.) [32, 33], the interspecific cross of *Q. robur* x European sessile oak (*Q. petraea* (Matt.) Liebl.) [34], European chestnut (*Castanea sativa* Mill.) [35], Chinese chestnut (*C. mollissima* Blume) [36, 37], and the interspecific cross of Chinese chestnut x American chestnut (*C. dentata* (Marsh.) Borkh.) [38]. In the most recent report of genetic mapping in oaks, an 8 k custom genotyping array was used to generate very dense maps using two intraspecific and two interspecific full-sib families of *Quercus robur* and *Quercus petraea* [39]. Prior to our study, no structured crosses and no genetic maps existed for any of the *Lobatae*.

Paleobotanical data suggest that *Quercus* and *Lobatae* sections of the genus *Quercus* diverged between 15 and 40 Mya [17]. Even though both sections of the genus have 12 haploid chromosomes, the genetic barrier between the sections is complete. Results from other tree genera show that the number of shared SNPs decreases as the phylogenetic distance between species increases, suggesting that a SNP array based on closely related species in the European roburoid oaks may not be sufficiently informative for genetic mapping in the new world *Lobatae* [40]. One approach to overcome this difficulty is to use the transcriptome sequence of one species to do exome capture of a distantly related species, then sequence the captured pieces for SNP discovery [41]. The exome capture approach provides genetic resources that are otherwise problematic in the typically huge genomes (20 to 40 Gb) of conifers [42]. In contrast, the genome sizes of diploid angiosperm trees are much smaller (usually < 1Gb) and reasonably well-conserved within genera [13]. Thus we anticipated that ddRADseq, a technology that does not require any existing genomic tools other than a suitable mapping population, would have the potential to be a broadly applicable, low-cost approach for genetic mapping in woody angiosperms.

## Methods

### Mapping population

SM1 and SM2 are the labels given to the two parents of the mapping population. The parent trees are located on the campus of Purdue University, approximately in the middle of the native range for this species. The species identity was verified by co-author Dr. Mark Coggeshall. Parentage analysis identified SM2 as the predominant pollen parent for our selected seed tree SM1 [43]. The full-sib progeny used for this investigation were naturally pollinated by SM2 in 2009, hand-picked from SM1 in 2010 (*Q. rubra* has a 2-year acorn), parentage-verified with gSSR and outplanted in 2011. The progeny and parents were propagated as replicated clones at the Horticulture and Agroforestry Research Center (HARC) in New Franklin, MO in 2013. Co-author Coggeshall collected voucher specimens for SM1 and SM2 in 2017 and deposited sun leaves and shade leaves specimens for each parent in the Greene-Nieuwland Herbarium (herbarium code NDG) at the University of Notre Dame. The voucher specimen codes are ND145625, ND145626, ND145627, and ND145628.

### DNA extraction and DNA marker development

DNA was initially extracted from the parents and the 2010 sibship using a previously reported modified CTAB protocol [44]. For RADseq the 2010 sibship and the two parents were re-extracted with Qiagen DNeasy® Plant Mini kits according to the manufacturer’s protocol. For the framework map, we developed new gSSRs from a *Q. rubra* library enriched for CA repeats (Genetic Information Services, Chatsworth, CA). Primers were designed using Primer3 v. 0. 4.0 [45]. We also designed primers for 454-sequenced *Q. rubra* EST-SSRs detected in the northern red oak tissue above ground (ROA) and northern red oak roots below ground (ROB) (http://hardwoodgenomics.org/content/de-novo-northern-red-oak-quercus-rubra-ro454v2). We tested all CA and GA repeat gSSRs previously reported for *Q. rubra* [46–48], EST-SSRs reported for the European pedunculate oak *Quercus robur* L. [32] and EST-SSRs reported for the Chinese chestnut *Castanea mollissima* [49]. Markers were retained if the parental alleles occurred in any of the five configurations informative for mapping in the F_1_ progeny of outcrossing parents [50].

### PCR amplification and genotyping

All PCR reactions were carried out in an Eppendorf thermal cycler with a 10 μl reaction mixture composed of 2 μl of DNA (10 ng/μl), 4 pmol of each forward and reverse primers, 25 mM MgCl_2_, 10 mM dNTP, 1 μl of 10× Mg free PCR reaction buffer, 1 μl of 4% BSA, 0.25 U/μl TaKaRa Taq™ (Takara Bio USA, Mountain View, California) and 3.5 μl of double distilled H_2_O. The PCR amplification profile consisted of initial denaturation at 94 °C for 2 min, 35 cycles of 94 °C for 30 s, annealing at a marker specific temperature for 30 s, then 72 °C for 45 s followed by 60 °C for 45 min and ending with a final extension at 72 °C for 10 min. Fluorescently labeled amplicons were size fractionated on an ABI 3730 XL genetic analyzer (Applied Biosystems, Foster City, CA) using GeneScan™ 400 HD ROX™ (Applied Biosystems) as internal size standard. Fragment length polymorphisms were scored using GeneMapper® v 4.0 (Applied Biosystems). Of the 379 SSR markers tested (67 gSSRs from *Q. rubra*, 180 EST-SSRs from *Q. rubra*, 120 bin-mapped EST-SSRs from *Q. robur* and 12 EST-SSRs from *C. mollissima*), 116 markers were informative (Additional file 1).

### ddRADseq library preparation and sequencing

We chose 225 full-sibs, a subset of the 399 full-sibs used for the framework map, for ddRADseq. Each DNA sample was diluted to a final concentration of 150 ng/μl and plated into a 96-well plate with each well containing 900 ng of DNA in a final volume of 6 μl. Library construction was done in the Genomics and Bioinformatics Core Facility at the University of Notre Dame. Libraries were prepared using a ddRADseq approach [51] modified for paired-end compatibility with additional modifications to size selection and library purification. Samples were digested with *Eco*RI and *Mse*I [51]. At the time the libraries were prepared, there were no publically released genomes for any of the Fagaceae. Thus we were not able to query a genome sequence to determine the optimum pair of restriction enzymes for producing fragment sizes appropriate for Illumina sequencing technology. Following restriction digestion, each sample was ligated with a unique indexed *Eco*RI adapter and an *Mse*I adaptor [52] modified for paired-end sequencing. Following ligation, samples were PCR amplified with iProof™ High-Fidelity DNA Polymerase (Bio-Rad, Hercules, California), pooled and purified using AMPure XP beads (Beckman Coulter Inc., Brea, CA) to make the ddRADseq library. In the final step, each library pool was size selected to a range of 300–500 bp using the BluePippin system (Sage Science Inc., Beverly, MA). Quantity and size distribution were assessed using the Qubit® 2.0 Fluorimeter (Life Technologies Corp., Carlsbad, CA) and Bioanalyzer 2100 System (Agilent Technologies, Santa Clara, CA).

Pooled libraries were sent to BGI International (Cambridge, MA) for sequencing. We pooled libraries of 50 full-sib samples per lane and to ensure accuracy of the SNP variant calls, used one lane for the two parent libraries with the expectation of obtaining better sequence coverage than the standard 96 samples per lane design. One of the progeny lanes contained libraries of 25 *Q. rubra* individuals not included in this project, so every progeny lane did have 50 individuals. Sequencing was done on an Illumina HiSeq 2000 using 101 bp paired-end reads.

### Preprocessing of raw reads

We checked raw sequences from the six Illumina HiSeq lanes for initial quality using FastQC [53]. As the forward reads were determined to be of high quality compared to the reverse reads, which were almost always much shorter, only the forward reads were used in the subsequent analyses [54, 55]. Adapters and poor quality sequences were removed using Trimmomatic [56] with recommended settings. Finally, reads were demultiplexed by index into separate libraries using a custom python script called trimmer [57]. This produced six files, one per lane, of FASTQ reads with sequence headers renamed to clearly indicate the individual from which they were derived.

### Generation of a *de novo* reference for Q. rubra

**Fig. 1:**
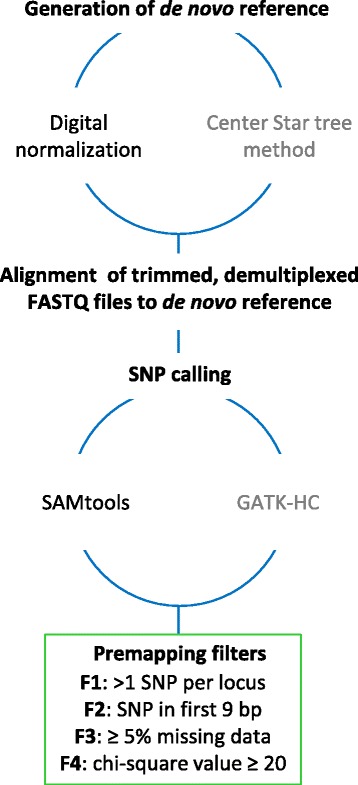
Summary of the bioinformatics pipeline for calling SNP variants

To minimize the computational requirements of deriving a reference assembly, we tested two non-alignment methods, a digital normalization and a center star method (Fig. 1). For the first method, we wrote a custom Perl script to perform “digital” normalization of ddRADseq data [58, 59]. First, a read was deemed a putative allele only if at least half of its 15-mers (15-mers are contiguous substrings of size 15 in a given read) were novel or not found in a previously saved allele. This has the benefit of being very simple to implement while removing repetitive sequences quickly. The downside of this approach is that low coverage alleles may still remain. To partially overcome this limitation we also aligned all 151.8 million reads using BWA [60] with less stringent settings (−k 3 -n 8) onto the putative loci. We then removed all potential alleles with four or fewer alignments along with extraordinarily highly covered alleles (>500 occurrences), most of which matched known oak plastid DNA.

For the center star method, progeny reads derived from either parent were clustered based on BWA alignments to a parental allele used to build a center star tree, such that the distance of each sequence is computed to all other sequences. The resulting reference set is all alleles that have the minimum distance to fellow progeny alleles, presumably because they have fewer sequencing errors. As the digital normalization strategy identified more hk x hk markers than the center star method, the digital normalization strategy was used for all subsequent steps (Additional file 2).

### Alignment of FASTQ files and SNP calling

The reads of the individual quality-controlled FASTQ files were aligned to the generated reference sequences using default parameters of BWA, SAMtools [61] and Picard tools (https://broadinstitute.github.io/picard/). These alignments were used to create the intermediate files for variant detection. We tested the SNP calling methods implemented in SAMtools and in the GATK HaplotypeCaller Walker annotated default [62], on both the digital normalization reference (DNR) and the center star reference (CSR). Finally, we set a filtration stage wherein only the SNPs for which the parents had an informative SNP configuration for mapping were retained. The digital normalization reference with SAMtools generated the most SNP calls (Additional file 2).

### Premapping filters

The informative SNP called by SAMtools were transformed into the JoinMap® format required for mapping with the F_1_ progeny of two outcrossing parents [50]. The premapping filters we tested first were missing data and the value of the chi-square test statistic for deviation from Mendelian expectation (F3 and F4, Fig. 1). We used two criteria to evaluate the effect of a range of cutoff values for missing data (0–30%) and the value of the chi-square test statistic (10–50). The first criterion was a reduction in map inflation, as determined by the difference in centimorgan length between the round two regression map and the maximum likelihood map for a given linkage group. In theory, if all the recombination events in the mapping population are detected, if no data are missing and if there are no genotyping errors, the regression map and the maximum likelihood map should be approximately the same length [63]. Our second criterion was preservation of the order of the markers on the framework map. We assumed that a low density framework map constructed with 399 full-sibs had sufficient statistical power to ascertain correct order in a diploid organism with 12 haploid chromosomes. These two criteria required that we generate maps using the two different mapping approaches (regression and maximum likelihood) for all of the linkage groups. Later, we added two additional premapping filters (F1 and F2, Fig. 1): the number of SNPs called within a given marker sequence and the position of the SNP within the sequence.

### Mapping

We generated the framework map first, using an independence LOD threshold of 20 for grouping markers and the Kosambi mapping function for regression mapping. For the final map, the initial data consisted of the 116 framework markers and 1413 SNP markers (see results for how our filters produced this number). Prior to mapping with the full dataset, we removed eight individuals with > 90% missing data (most of which were SNP markers), leaving a mapping population of 217 individuals. Finally, we excluded SNP markers with similarity value ≥ 0.945, leaving 1344 unique SNP markers and all of the framework markers. The data were grouped using an independence LOD threshold of 30. Framework markers were specified using the fixed order function in JoinMap® 4.1 before mapping. The fixed order function specifies only a fixed order, not a fixed distance. We generated maps using both the approximate maximum likelihood and regression mapping algorithms with default settings. The final map was generated using round two regression mapping with the Kosambi mapping function and charted using MapChart 2.30 [64].

### Downsampling experiments

We tested the effectiveness of our conservative use of sequencing capacity (one lane for the two parents and only 50 progeny per lane) by comparing our results with those we may have obtained if we had used a less conservative design. We conducted two progeny downsampling experiments (Exp1 and Exp2). For Exp1, we downsampled the trimmed and demultiplexed FASTQ files for the 225 progeny used for mapping while keeping the parent data intact, to simulate 96 progeny per lane, but reserving one lane for the two parents. For Exp2, we downsampled both progeny and parents to simulate the condition in which all of the progeny and the parent libraries were run in three lanes: 96 progeny samples in two lanes, 33 progeny in the third lane with 31 replicates for one parent and 32 for the other.

We implemented the downsampling approach by randomly selecting 52% (50/96 *100) of the FASTQ data from each progeny for the downstream analysis. For Exp2, progeny downsampling was the same as in Exp1. For the parents, our design has the effect of utilizing ~32% of the sequencing lane capacity for each parent, as opposed to 50% based on our initial sequencing approach. We see this as a choice an investigator is likely to make, to save the cost of using another lane, while at the same time getting more parent reads. We implemented parent downsampling by randomly selecting 64% (32/50*100) of the data for each parent for downstream analysis. In all experiments, we used the DNR approach, with SAMtools as the SNP caller, the same as we did with the full data set.

## Results

### SNP calling approach and premapping filtration

The number of reads for parents and progeny totaled 877,796,304. The parent lane yielded 88,788,165 reads for SM1 and 61,994,649 reads for SM2. The mean number of reads per progeny was 2,908,053, the median 3,056,516. Using the DNR reference sequence, SAMtools called more than six times as many SNPs (78,725) as Haplotype Caller (12,694) (Additional file 2). Both SNP callers produced far fewer SNP calls with the CSR reference sequence. We chose to proceed with the 78,725 SNPs called by SAMtools.

Our initial filters (missing data and value of the chi-square test statistic) resulted in severely inflated maps, even at the strict criteria of 5% missing data and a chi-square value < 10. The maximum likelihood map for linkage group 3 exceeded 1000 cM, nearly ten times the distance inferred with the round two regression map. All other linkage groups were inflated as well. We found that sequences with > 1 SNP call were driving this result. Of the 78,725 ddRADseq markers in which informative SNPs were detected, 60,687 (77%) had > 1 SNP. These 60,687 SNPs occurred on 21,526 ddRADseq sequences, indicating that some sequences had more than two SNPs. Our query of Repbase (http://www.girinst.org/repbase/) for matches to the 21,526 sequences with > 1 SNP resulted in only 46 matches at an E-value ≤ 9.91E-07, 34 of which had best hits to Gypsy or Copia LTR-retrotransposons. Our query of P-mite, a database for plant miniature inverted-repeat transposable elements [65] yielded only two good alignments. We suspect that our query sequences may be too short (~80–120 bp) for accurate, strong annotations and that the repeats in *Q. rubra* may have diverged in sequence significantly from the repeats of the model plants represented in the two databases. Removal of these multi-SNP loci left 18,038 SNP markers. Finally, previous experience with SNP chips suggests that SNPs located in the first 9 bp of the marker sequence are more likely to generate artifacts. After removal of loci with SNPs in the first 9 bp (2263 markers), 15,775 SNP markers remained for use in mapping. This filtered SNP marker number is greater than the number of unfiltered SNP calls we obtained with GATK-HaplotypeCaller (12,694 SNPs).

### Final filtration

As both algorithms we used for genetic mapping are sensitive to missing data [63], we tested three conservative filters for missing data (none, 2%, and 5%) on the remaining set of 15,775 SNP markers. For each level of missing data, we generated chi-square cutoff values of 50 and 20. The number of markers remaining at a chi-square cutoff of 20 was only slightly smaller than the number remaining at a chi-square cutoff of 50 at each level of missing data tested. The final set of 1413 SNP markers used for mapping had < 5% missing data and a chi-square value < 20. This number was further reduced to 1344 after removal of markers with highly similar or identical genotypes (≥0.945).

### Final map construction

**Fig. 2:**
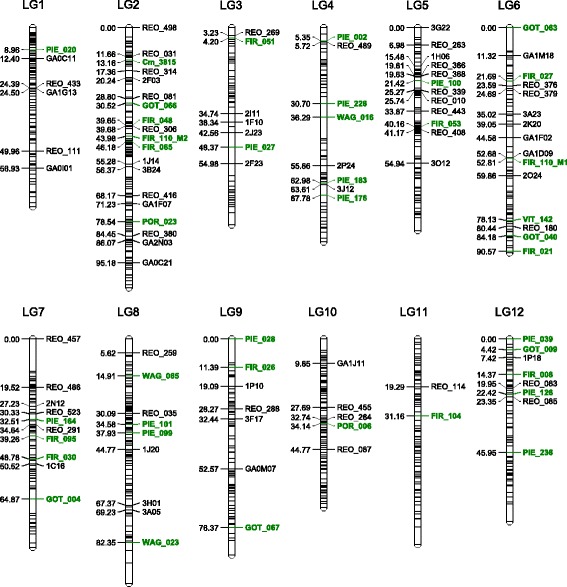
Genetic linkage map for *Q. rubra*. Linkage groups shown to scale in cM. Unlabeled *black* bars indicate SNP positions. The REO prefix indicates an EST-SSR from *Q. rubra*. The GA prefix indicates a GA repeat gSSR from *Q. rubra*. A prefix beginning with a number indicates a CA repeat gSSR from *Q. rubra*. Markers having labels in bold *green* type are EST-SSR from *Q. robur*. Linkage groups have the same numbers as those given to the *Q. robur* linkage groups

The framework map identified 12 linkage groups containing a total of 108 SSR markers. Eight of the 116 SSR markers were excluded during the mapping process. The round 3 framework regression map spanned a total length of 652.2 cM with an average spacing between markers of 6 cM. The map included 39 *Q. robur* EST-SSR markers across the 12 *Q. rubra* linkage groups. In those linkage groups with three or more *Q. robur* markers (2, 4, 6, 7, 8, 9, 12), the order is the same (Fig. 2) as that previously reported for the *Q. robur* maps [32]. Based on this initial evidence for colinearity, we have given our linkage groups the same numbers as those given to the *Q. robur* linkage groups.

GO annotations suggested stress resistance functions for three of the 73 EST-SSRs located on the final map. The PIE_126 sequence matches the *Quercus robur* cDNA clone LG0AAA8YO09RM1 (NCBI FP025018). The GO annotation suggests similarity to a family of proteins involved in response to cadmium stress [66]. The WAG_023 sequence matches the *Quercus petraea* cDNA clone WZ0AQPAI7YG19FM1 (NCBI FN736994). The GO annotation suggests similarity to *Arabidopsis* genes involved in response to colder temperatures [67]. The FIR_008 sequence matches *Quercus robur* cDNA clone LG0AAA8YO09RM1 (NCBI FP025018). The GO annotation suggest similarity to Calcineurin B-like protein 9, a protein involved in the regulation of early stress-related CBF/DREB transcription factors [68].

To generate the final map we used the 217 individuals that had both framework markers and high-quality SNP marker genotypes. Our round two final regression map contains 957 markers distributed over 1014.47 cM (Table 1, Fig. 2). The mean read depth in the parents for the SNPs in the final map was 248× (median 241×). The mean read depth in the progeny for this final set of SNPs was 13× (median 14.8×).

**Table 1:** Summary description of the *Q. rubra* map

Linkage Group	# Loci	*Q. robur* EST-SSR markers	Length (cM)	Density^a^
LG1	94	1	72.5	0.77
LG2	122	5	105.4	0.86
LG3	51	2	79.9	1.56
LG4	59	5	86.8	1.47
LG5	88	2	82.2	0.93
LG6	70	6	91.2	1.30
LG7	71	4	84.4	1.18
LG8	98	4	99	1.01
LG9	71	3	87.5	1.23
LG10	85	1	68.3	0.80
LG11	79	1	83.3	1.05
LG12	69	5	73.9	1.07
Total	957	39	1014.4	

^a^Average number of markers/cM

The longest linkage groups (LG2 and LG8) on the *Q. robur*-*Q. petraea* consensus map [39] were also the longest linkage groups on the *Q. rubra* map. The areas in the *Q. rubra* linkage groups in which SNPs are markedly absent were the regions where the sequences with >1 SNP were concentrated, especially on LG3, where the map inflation was the most severe if these sequences were included. We found no evidence for segregation distortion in any of these 957 markers using the method described by Bodénès et al. [39] for the *Q. robur*-*Q. petraea* consensus map. The *Q. robur* EST marker FIR_110 produced two different sets of informative alleles, mapping to LG6 and LG2 (Fig. 2, Additional files 1 and 3). The marker FIR_110 maps to LG6 in *Q. robur* [32].

### Downsampling results

In Exp1, the trimmed and demultiplexed FASTQ files for the progeny were downsampled, while the parent FASTQ files were not downsampled. This yielded 5090 SNP calls using DNR and SAMtools. When both the parents and the progeny were downsampled in Exp2, the yield was even smaller (1616 SNP calls) (Table 2). Using the filtering criteria we used to construct the map reported here, only six SNP markers remained after Exp1 downsampling and three SNP markers for Exp2. If we had used the less strict criteria of < 10% missing data with a chi-square cutoff of 20 on the Exp1 data, 61 SNP markers would have remained. Given that the point of using ddRADseq is to produce enough SNP markers for a dense map, this reduced depth of sampling produces an unsatisfactory result. Given the necessity of generating a *de novo* reference and our goal of generating a dense map, the sequencing design we actually used (allocating an entire lane to the two parents and multiplexing 50 progeny per lane) proved to be an effective one.

**Table 2:** SNP calls remaining after sequential filtration of downsampling experiment data

Sequential filtration	Exp1	Exp2
None	5090	1616
After F1^a^	4500	1449
After F2^b^	4055 (2304)^c^	1343 (545)^c^
After MD filter >20%^d^	550 (347)^c^	320 (132)^c^
After MD filter >10%^d^	113 (61)^c^	63 (20)^c^
After MD filter ≥5%^d^	20 (6)^c^	10 (3)^c^

^a^Removal of markers with >1 SNP in the same sequence

^b^Removal of markers in which the SNP occurs in the first 9 bases of sequence

^c^Subset of markers meeting criterion of chi-square value ≤ 20

^d^Removal of markers in which >20%, >10% or ≥5% of 217 individuals have missing data, as indicated

## Discussion

Any technical advance that puts genomics technology within the reach of those who work on non-model systems tends to be quickly embraced with great enthusiasm, followed by a more measured approach once technical limitations are understood. This is certainly the case with RADseq. Our purpose in this study was twofold: 1) a rigorous test of the ddRADseq approach for constructing dense genetic maps in outcrossing, undomesticated woody perennials lacking a reference genome and 2) the production of a high-quality linkage map for *Q. rubra*, the most widely distributed species in the speciose *Lobatae* section of the *Quercus* genus. Our study design enabled us to examine the effects of lower coverage, alignment methods, and variant callers on the yield of SNP markers suitable for high quality genetic mapping in an organism lacking a reference genome. The second step in genetic mapping is grouping and inference of linear order, a process which requires a sound understanding of the limits of statistical inference in genetic mapping.

### Inferred linear order vs. the actual linear order

Genetic mapping projects have the advantage of two of the strongest priors in all of biology: Mendelian expectation and the linear information storage system of DNA. The first prior enables a rigorous test of the performance of a RRS technology and associated informatics pipelines for accurate and consistent detection of alleles, i.e. alleles present in the parents, if correctly called, must be present in the progeny of these parents and will occur with an expected frequency in the progeny population. Next, the probability of recombination between any two loci in the linear DNA array is a function of the distance between them. Finally, if the variant calls are correct and the recombination estimates are accurate, then the inferred linear order of the markers will be the actual linear order if the inference algorithm is appropriate.

In genotyping by sequencing, the requirement for accurate calls is likely to be met by high coverage, but the depth of sequencing coverage for parent and for progeny need not be equal, as we have shown. Our downsampling experiment indicated that multiplex sequencing 96 samples per lane would not have produced enough high-quality data for genetic mapping, even if the parents were sequenced to a high depth of coverage. Thus the answer to the design problem of “large numbers of individuals at low depth vs. a small number of individuals at greater depth” has different solutions depending on the intended use. When no reference genome is available, generating good sequencing depth in the progeny (to ensure consistency of SNP variant calls) and higher sequencing depth in the parents (to ensure accuracy of the SNP variant calls) is prudent, regardless of other conditions. The values of “good” and “higher” can be approximated by *in silico* digests of a related genome, but at the time this project was designed there were no genomes released for any oak species. With the *Q. robur* and *Q. lobata* (*Quercus* section Quercus) genomes now released [69, 70] the number and size of the cut sites, as well as the optimum combination of restriction enzymes may be estimated and the project design adjusted accordingly.

The primary purpose of our work was to generate a high quality genetic map and, by using sequenced markers, provide a tool for correctly ordering sequence scaffolds and contigs to chromosomal locations. However, a given progeny population contains a fixed amount of information regardless of the marker system used for detection, whereas mapping algorithms have no limit on map length. Thus the measure of map quality must not be how many of the SNPs called were mapped. If the LOD criteria used for grouping and mapping are low and very similar SNP genotypes are included, longer linkage groups may result, but the relationship of this inference to actual order may be weak. The “ground truth” test of comparing the inferred linear order with the actual linear order is rarely available for non-model organisms. A useful indirect test is a comparison of the map length produced by a regression approach with that produced by approximate maximum likelihood. This approach is well described by others [50, 63], but given the surge in genetic mapping projects made possible by RSS technologies, it is useful to point out here that a regression approach is designed to reject loci for poor fit (e.g. a locus that produces negative distance estimates). A maximum likelihood approach has the requirement of accounting for all of the markers in the group. Mathematically, this requires that the overall map distance must lengthen to accommodate the most poorly fitting markers. This is a major source of map inflation if many markers fit poorly. Thus a comparatively quick indirect check on the quality of a map is a comparison of the length of the regression map to the length of the maximum likelihood map, for each linkage group. If genetic maps are to be useful for ordering scaffolds and for gene discovery, some measure of quality control is essential.

### *De novo* reference genomes from ddRADseq data

When a reference genome is lacking, one must be generated *de novo* from the RADseq data itself. Our initial tests indicated that our digital normalization approach, with the SAMtools variant caller, yielded the most SNP calls (78,725). A recent investigation of the accuracy of variant calling pipelines across different technology platforms showed that a variation of the BWA alignment tool (BWA-MEM) with the SAMtools SNP variant caller, performed better on Illumina data than the BWA-MEM with the GATK-HC pipeline [71]. This suggests that our BWA-SAMtools pipeline actually did detect more real SNPs than the BWA-GATK-HC pipeline. However, after additional filtration and mapping, only 849 of 78,725 SNPs variants detected (1.8%) were placed on our map. Most of the SNP variants (77%) were rejected for having >1 SNP in the sequence. We suspect that these SNPs were accurately called but occur in sequences in different places within linkage groups, violating the necessary assumption that the SNP variants detected are alleles of a single locus. This violation would generate the huge map inflation we observed. Our results are consistent with the results of a recent study in which a reference genome was available [72]. Zhang et al. found that of the three references tested (unmasked scaffolds, repeat masked scaffolds, and gene models), the repeat masked genome produced the best map. The percentage of ddRADseq markers anchored to the top 10 megascaffolds was highest with markers detected using repeat masked scaffolds. Many of the markers detected using unmasked scaffolds were present on more than one scaffold, while markers detected using only gene models are too few to generate a dense map. Using restriction enzymes that target sites within the gene space would minimize the number of SNPs detected in repeated sequences, but reduce the utility of the resulting low coverage map for ordering contigs from whole genome sequencing projects.

### The 5% standard for missing data

Even with the aid of a good framework map and using only those SNPs remaining after application of the premapping filters, we found that map inflation was best minimized using the strict criterion of 5% missing data, a lower value than the 10–25% typically reported for genetic maps constructed with RRS technologies [3, 31, 73, 74]. Improvements in sequencing technologies and methods of library construction could address the problem of missing data, as long as the calling accuracy (the number of times a variant is detected when it is present) is part of the quality control process. Mapping populations of full-sib progeny from the same two parents in a long-lived forest tree species provide an excellent source of positive controls for such technology improvements.

### Mapping with haploid tissues

Regardless of the technology employed, the construction of a high-quality genetic map using the F_1_ progeny of outcrossing, highly heterozygous parents is an exacting and tedious process. In conifers, genetic maps can be constructed using the haploid megagametophyte seed storage tissue from the seeds of a single tree [75–77], as was recently done with ddRADseq for the white cypress pine, *Callitris glaucophylla* [78]. The technology for genotyping single pollen grains, the only easily accessible haploid tissue in angiosperms, exists [79] and was recently demonstrated in hyūganatsu (*Citrus tamurana*) [80], but the technical challenges are considerable. For undomesticated angiosperm forest trees, especially the ecologically dominant, economically valuable and speciose oaks, the only feasible method at present is to use the progeny of known parents.

## Conclusions

Using ddRADseq in combination with an SSR-based framework map, we have constructed an oak genetic map that will enable testing of explicit hypotheses about the organization of loci contributing to adaptive evolution in oaks and provide a tool for the detection of allelic variants contributing to stress tolerance. Although mapping stress tolerance genes was not the main focus of this study, three of the 73 EST-SSR markers located on the final map have annotations suggestive of involvement in stress tolerance. The generation of a moderately dense genetic map in *Q. rubra* complements the dense map produced for the European oaks *Q. robur* and *Q. petraea* [39], confirms synteny and provides evidence of high colinearity across two genetically incompatible sections of the *Quercus* genus. These dense maps, together with the data from the *Q. robur* genome, the *Q. lobata* (California valley oak) genome [70] and the *Castanea mollissima* (Chinese chestnut) genome [36, 37, 81, 82], will greatly foster our understanding of the genetic architecture of the genus *Quercus* and of the Fagaceae (oaks, chestnuts and beeches), a major family of forest trees in the temperate and subtropical regions of the world. Finally, we anticipate that improved, low-cost RRS technologies and more accessible informatics pipelines will enable the solution of a fundamental puzzle in evolutionary biology, one for which oaks are justifiably famous: rapid sympatric speciation, in the presence of persistent gene flow, within and across a wide array of ecological niches, on all of the continents in which the oaks are native.

## Additional files

Additional file 1: All informative microsatellite markers and associated data. In total 116 informative markers were developed for *Q. rubra*. These include 37 gSSRs, 38 EST-SSRs from *Q. rubra*, 1 *C. mollissima* EST-SSR and 40 *Q. robur* EST-SSRs. The table shows the marker name, the corresponding sequence accession numbers from NCBI, forward and reverse primers used for amplification and base pair sizes in *Q. rubra* mapping parents. (XLSX 78 kb)

Additional file 2: Performance of SNP variant callers with two methods of *de novo* reference construction. Tabular data shows how the number of SNP markers generated by SAMtools and HaplotypeCaller changed after elimination of the SNPs with 30% or more missing genotypes for each of the three marker categories possible with SNPs and informative for mapping in F_1_ of outcrossing parents. (XLSX 9 kb)

Additional file 3: *Q. rubra* mapped markers and associated data. The table shows all of the mapped framework and SNP markers, the cM distances between them, marker category, position on the *Q. rubra* linkage group, the sequence in which the marker occurs and the genotypes for the 217 full-sib progeny used for mapping. (XLSX 890 kb)

## Abbreviations

CSR: Center star reference
ddRADseq: Double digest restriction site associated DNA sequencing
DNR: Digital normalization reference
RADseq: Restriction site associated DNA sequencing
RRS: Reduced representation sequencing
SNP: Single nucleotide polymorphism
